# Redetermination of 2,2′-bipyridine-1,1′-diium dibromide

**DOI:** 10.1107/S1600536812040214

**Published:** 2012-09-29

**Authors:** Basem F. Ali, Rawhi Al-Far, Salim F. Haddad

**Affiliations:** a Department of Chemistry, Al al-Bayt University, Mafraq 25113, Jordan; b Faculty of Science and IT, Al-Balqa’a Applied University, Salt, Jordan; c Department of Chemistry, The University of Jordan, Amman 11942, Jordan

## Abstract

In the title mol­ecular salt, C_10_H_10_N_2_^2+^·2Br^−^, the dihedral angle between the aromatic rings is 20.83 (14)° and the N—H groups have a *transoid* conformaton [N—C—C—N = 158.5 (3)°]. In the crystal, the cations are linked to the anions by two N—H⋯Br and five C—H⋯Br hydrogen bonds, generating corrugated sheets incorporating *R*_2_^1^(7), *R*_4_^2^(10), *R*_4_^2^(11) and two different *R*_4_^2^(12) loops. This structure was originally reported by Nakatsu *et al.* [Acta Cryst (1972), A**28**, S24], but no atomic coordinates are available.

## Related literature

For the previous report of this structure as a conference abstract, see: Nakatsu *et al.* (1972 ▶). For related structures of 2,2′-bipyridium dication salts, see: Ma *et al.* (2000 ▶). For structures containing dicationic 2,2′-bipyridyl derivative salts, see: Amarante *et al.* (2011 ▶); Eckensberger *et al.* (2008 ▶). For structures of monocationic 2,2′-bipyridinium salts, see: Kavitha *et al.* (2006 ▶). For ring motifs, see: Bernstein *et al.* (1995 ▶).
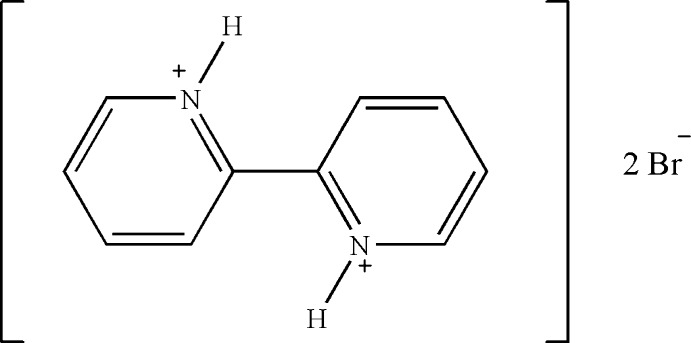


## Experimental

### 

#### Crystal data

C_10_H_10_N_2_^2+^·2Br^−^
*M* *_r_* = 318.00
Monoclinic, 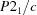

*a* = 7.5568 (6) Å
*b* = 9.7747 (7) Å
*c* = 15.3533 (12) Å
β = 95.830 (7)°
*V* = 1128.21 (15) Å^3^
*Z* = 4
Mo *K*α radiation
μ = 7.15 mm^−1^
*T* = 293 K
0.20 × 0.15 × 0.10 mm

#### Data collection

Agilent Xcalibur EOS diffractometer
Absorption correction: multi-scan (*CrysAlis PRO*; Agilent, 2011 ▶) *T*_min_ = 0.287, *T*_max_ = 0.489
5425 measured reflections
3054 independent reflections
1884 reflections with *I* > 2σ(*I*)
*R*_int_ = 0.032

#### Refinement

*R*[*F* ^2^ > 2σ(*F* ^2^)] = 0.037
*wR*(*F* ^2^) = 0.073
*S* = 1.03
3054 reflections
127 parameters
H-atom parameters constrained
Δρ_max_ = 0.50 e Å^−3^
Δρ_min_ = −0.57 e Å^−3^

### 

Data collection: *CrysAlis PRO* (Agilent, 2011 ▶); cell refinement: *CrysAlis PRO*; data reduction: *CrysAlis PRO*; program(s) used to solve structure: *SHELXS97* (Sheldrick, 2008 ▶); program(s) used to refine structure: *SHELXL97* (Sheldrick, 2008 ▶); molecular graphics: *SHELXTL* (Sheldrick, 2008 ▶); software used to prepare material for publication: *SHELXTL*.

## Supplementary Material

Crystal structure: contains datablock(s) I, global. DOI: 10.1107/S1600536812040214/hb6957sup1.cif

Structure factors: contains datablock(s) I. DOI: 10.1107/S1600536812040214/hb6957Isup2.hkl

Supplementary material file. DOI: 10.1107/S1600536812040214/hb6957Isup3.cml

Additional supplementary materials:  crystallographic information; 3D view; checkCIF report

## Figures and Tables

**Table 1:** Hydrogen-bond geometry (Å, °)

*D*—H⋯*A*	*D*—H	H⋯*A*	*D*⋯*A*	*D*—H⋯*A*
N1—H1*A*⋯Br2	0.86	2.37	3.178 (3)	156
N2—H2*A*⋯Br1^i^	0.86	2.35	3.145 (3)	154
C1—H1*B*⋯Br1	0.93	2.83	3.611 (4)	143
C9—H9*A*⋯Br2	0.93	2.81	3.675 (4)	155
C6—H6*A*⋯Br2^i^	0.93	2.84	3.619 (4)	142
C4—H4*A*⋯Br1^i^	0.93	2.86	3.697 (4)	150
C3—H3*A*⋯Br1^ii^	0.93	2.85	3.677 (4)	149

Symmetry codes: (i) 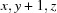; (ii) 
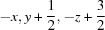
.

**Fig. 1.**
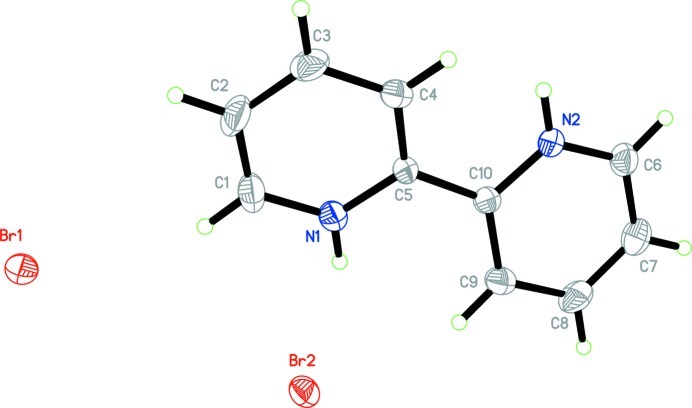
The molecular stucture of the title salt with displacement ellipsoids drawn at the 30% probability level.

**Fig. 2.**
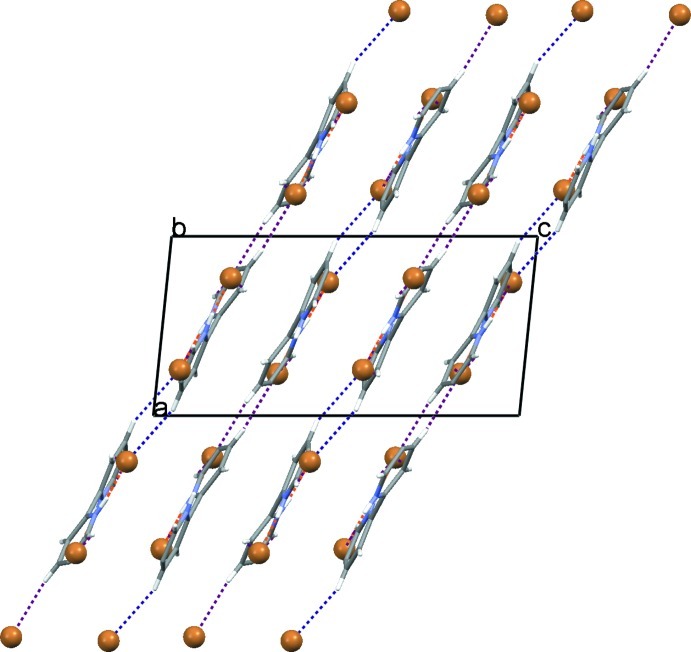
Packing diagram of the title salt showing the sheets of cations···anions hydrogen bonded species. Interspecies C/N–H···Br hydrogen bonds are shown as dashed lines. H atoms have been omitted for clarity, except for those involved in hydrogen bonds (shown as dashed lines).

**Fig. 3.**
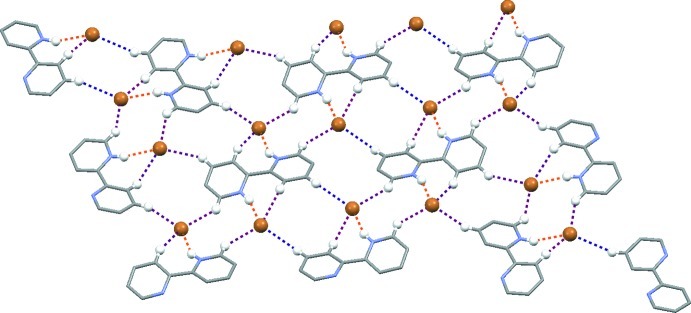
A view of one hydrogen bonded sheet contains the different graph-set motifs in the title salt.

## supplementary crystallographic information

### Comment

2,2'-bipyridine can form two types of salts monocationic, see for example (Kavitha *et al.*, 2006) or dicationic bipyridinium (see for example Ma *et al.*, 2000). Herein we report the title salt, (I), Figure 1. This structure was reported by Nakatsu *et al.* (1972), but no atomic coordinates are available. The bipyridinium cation in (I) has a *transoid* configuration with the N—C—C—N torsion angle is 158.5 (3)°, and the C—C bond distance between the two rings being 1.464 (4) Å. Within the dication, geometrical dimensions are in normal range and agree with reported values (Ma *et al.*, 2000; Eckensberger *et al.* 2008; Amarante *et al.* 2011).

The ions in (I) are linked by a combination of seven hydrogen bonds of the types N—H···Br and C—H···Br, Table 1, into complex corrugated sheets, Figure 2. These sheets composed of *R^1^_2_(7), R^2^_4_(10), R^2^_4_(11) and two different R^2^_4_(12)* graph set motifs (Bernstein *et al.* 1995), Figure 3. With the overall coordination around each bromide anion being 4 (three C—H···Br and one N—H···Br interactions), Figure 3.

### Experimental

A solution of MnCl_2_ (0.1258 g, 1 mmol) dissolved in 95% EtOH (10 ml) solution was added to a mixture of 2,2'-bipyridine (0.1562 g, 1 mmol) dissolved in 95% EtOH (10 ml) and 60% HBr (2 ml). The resulting mixture was heated to few min, then treated with molecular bromine (2–3 drops), then refluxed for 1.5 hr. On slow evaporation at room temperature colourless chunks of the title salt were formed.

### Refinement

All H atoms were positioned geometrically and refined using a riding model, with N—H = 0.86 Å and C—H = 0.93 Å, with the *U*_iso_(H) were allowed at 1.2*U*_eq_(N/C).

### Crystal data

**Table:**

C_10_H_10_N_2_^2+^·2Br^−^	*F*(000) = 616
*M**_r_* = 318.00	*D*_x_ = 1.872 Mg m^−^^3^
Monoclinic, *P*2_1_/*c*	Mo *K*α radiation, λ = 0.71073 Å
Hall symbol: -P 2ybc	Cell parameters from 1832 reflections
*a* = 7.5568 (6) Å	θ = 2.9–29.1°
*b* = 9.7747 (7) Å	µ = 7.15 mm^−^^1^
*c* = 15.3533 (12) Å	*T* = 293 K
β = 95.830 (7)°	Chunk, colourless
*V* = 1128.21 (15) Å^3^	0.20 × 0.15 × 0.10 mm
*Z* = 4	

### Data collection

**Table:**

Agilent Xcalibur EOS diffractometer	3054 independent reflections
Radiation source: Enhance (Mo) x-ray source	1884 reflections with *I* > 2σ(*I*)
Graphite monochromator	*R*_int_ = 0.032
Detector resolution: 16.0534 pixels mm^-1^	θ_max_ = 29.2°, θ_min_ = 3.4°
ω scans	*h* = −7→10
Absorption correction: multi-scan (*CrysAlis PRO*; Agilent, 2011)	*k* = −13→10
*T*_min_ = 0.287, *T*_max_ = 0.489	*l* = −21→16
5425 measured reflections	

### Refinement

**Table:**

Refinement on *F*^2^	Primary atom site location: structure-invariant direct methods
Least-squares matrix: full	Secondary atom site location: difference Fourier map
*R*[*F*^2^ > 2σ(*F*^2^)] = 0.037	Hydrogen site location: inferred from neighbouring sites
*wR*(*F*^2^) = 0.073	H-atom parameters constrained
*S* = 1.03	*w* = 1/[σ^2^(*F*_o_^2^) + (0.025*P*)^2^] where *P* = (*F*_o_^2^ + 2*F*_c_^2^)/3
3054 reflections	(Δ/σ)_max_ = 0.001
127 parameters	Δρ_max_ = 0.50 e Å^−^^3^
0 restraints	Δρ_min_ = −0.57 e Å^−^^3^

### Special details

**Table:**

Geometry. All e.s.d.'s (except the e.s.d. in the dihedral angle between two l.s. planes) are estimated using the full covariance matrix. The cell e.s.d.'s are taken into account individually in the estimation of e.s.d.'s in distances, angles and torsion angles; correlations between e.s.d.'s in cell parameters are only used when they are defined by crystal symmetry. An approximate (isotropic) treatment of cell e.s.d.'s is used for estimating e.s.d.'s involving l.s. planes.
Refinement. Refinement of *F*^2^ against ALL reflections. The weighted *R*-factor *wR* and goodness of fit *S* are based on *F*^2^, conventional *R*-factors *R* are based on *F*, with *F* set to zero for negative *F*^2^. The threshold expression of *F*^2^ > σ(*F*^2^) is used only for calculating *R*-factors(gt) *etc*. and is not relevant to the choice of reflections for refinement. *R*-factors based on *F*^2^ are statistically about twice as large as those based on *F*, and *R*- factors based on ALL data will be even larger.

### Fractional atomic coordinates and isotropic or equivalent isotropic displacement parameters (Å^2^)

**Table:**

	*x*	*y*	*z*	*U*_iso_*/*U*_eq_	
Br1	0.23397 (5)	0.03486 (4)	0.67326 (2)	0.04534 (13)	
N1	0.4480 (4)	0.4858 (3)	0.63133 (16)	0.0384 (7)	
H1A	0.5366	0.4580	0.6051	0.046*	
C1	0.3257 (6)	0.3947 (4)	0.6491 (2)	0.0507 (10)	
H1B	0.3369	0.3036	0.6330	0.061*	
N2	0.5392 (4)	0.8449 (3)	0.61771 (15)	0.0369 (7)	
H2A	0.4378	0.8714	0.6323	0.044*	
C2	0.1841 (6)	0.4356 (4)	0.6909 (2)	0.0554 (11)	
H2B	0.0950	0.3739	0.7013	0.066*	
Br2	0.74405 (5)	0.29766 (4)	0.55966 (2)	0.04749 (13)	
C3	0.1749 (5)	0.5695 (4)	0.7175 (2)	0.0532 (11)	
H3A	0.0822	0.5979	0.7488	0.064*	
C4	0.3033 (5)	0.6619 (4)	0.6978 (2)	0.0429 (9)	
H4A	0.2968	0.7526	0.7154	0.051*	
C5	0.4402 (4)	0.6197 (3)	0.65239 (19)	0.0314 (7)	
C6	0.6477 (5)	0.9395 (4)	0.5912 (2)	0.0446 (9)	
H6A	0.6141	1.0311	0.5898	0.054*	
C7	0.8083 (6)	0.9019 (4)	0.5662 (2)	0.0505 (10)	
H7A	0.8851	0.9671	0.5470	0.061*	
C8	0.8553 (5)	0.7657 (4)	0.5698 (2)	0.0509 (10)	
H8A	0.9641	0.7383	0.5524	0.061*	
C9	0.7398 (5)	0.6683 (4)	0.5997 (2)	0.0449 (9)	
H9A	0.7723	0.5765	0.6035	0.054*	
C10	0.5767 (4)	0.7105 (3)	0.62330 (19)	0.0324 (8)	

### Atomic displacement parameters (Å^2^)

**Table:**

	*U*^11^	*U*^22^	*U*^33^	*U*^12^	*U*^13^	*U*^23^
Br1	0.0395 (2)	0.0378 (2)	0.0598 (2)	0.00431 (18)	0.01032 (17)	−0.00318 (18)
N1	0.0397 (19)	0.0320 (17)	0.0441 (16)	−0.0004 (14)	0.0068 (13)	0.0021 (13)
C1	0.058 (3)	0.035 (2)	0.058 (2)	−0.013 (2)	0.001 (2)	0.0056 (18)
N2	0.0309 (17)	0.0316 (17)	0.0483 (17)	−0.0033 (14)	0.0055 (13)	0.0014 (13)
C2	0.043 (3)	0.056 (3)	0.067 (3)	−0.019 (2)	0.008 (2)	0.016 (2)
Br2	0.0500 (3)	0.0356 (2)	0.0580 (2)	0.00351 (19)	0.01070 (18)	−0.00453 (16)
C3	0.036 (2)	0.060 (3)	0.067 (2)	0.004 (2)	0.0223 (19)	0.018 (2)
C4	0.037 (2)	0.036 (2)	0.057 (2)	0.0052 (17)	0.0111 (18)	0.0031 (17)
C5	0.0301 (19)	0.0260 (18)	0.0379 (17)	−0.0007 (16)	0.0022 (14)	0.0036 (14)
C6	0.045 (3)	0.037 (2)	0.052 (2)	−0.0067 (19)	0.0020 (18)	0.0054 (17)
C7	0.048 (3)	0.052 (3)	0.053 (2)	−0.015 (2)	0.0113 (19)	0.0052 (19)
C8	0.034 (2)	0.065 (3)	0.056 (2)	−0.005 (2)	0.0157 (17)	−0.002 (2)
C9	0.038 (2)	0.038 (2)	0.060 (2)	0.0053 (18)	0.0112 (18)	0.0026 (17)
C10	0.0304 (19)	0.0319 (19)	0.0349 (17)	0.0001 (16)	0.0033 (14)	0.0023 (14)

### Geometric parameters (Å, º)

**Table:**

N1—C1	1.331 (4)	C4—C5	1.369 (4)
N1—C5	1.351 (4)	C4—H4A	0.9300
N1—H1A	0.8600	C5—C10	1.464 (4)
C1—C2	1.363 (6)	C6—C7	1.360 (5)
C1—H1B	0.9300	C6—H6A	0.9300
N2—C6	1.326 (4)	C7—C8	1.378 (5)
N2—C10	1.345 (4)	C7—H7A	0.9300
N2—H2A	0.8600	C8—C9	1.399 (5)
C2—C3	1.375 (5)	C8—H8A	0.9300
C2—H2B	0.9300	C9—C10	1.383 (5)
C3—C4	1.382 (5)	C9—H9A	0.9300
C3—H3A	0.9300		
			
C1—N1—C5	123.5 (3)	N1—C5—C4	117.8 (3)
C1—N1—H1A	118.3	N1—C5—C10	117.7 (3)
C5—N1—H1A	118.3	C4—C5—C10	124.4 (3)
N1—C1—C2	119.6 (4)	N2—C6—C7	119.7 (4)
N1—C1—H1B	120.2	N2—C6—H6A	120.1
C2—C1—H1B	120.2	C7—C6—H6A	120.1
C6—N2—C10	124.6 (3)	C6—C7—C8	118.9 (4)
C6—N2—H2A	117.7	C6—C7—H7A	120.6
C10—N2—H2A	117.7	C8—C7—H7A	120.6
C1—C2—C3	119.0 (4)	C7—C8—C9	120.3 (4)
C1—C2—H2B	120.5	C7—C8—H8A	119.9
C3—C2—H2B	120.5	C9—C8—H8A	119.9
C2—C3—C4	119.9 (4)	C10—C9—C8	119.0 (3)
C2—C3—H3A	120.0	C10—C9—H9A	120.5
C4—C3—H3A	120.0	C8—C9—H9A	120.5
C5—C4—C3	120.0 (3)	N2—C10—C9	117.6 (3)
C5—C4—H4A	120.0	N2—C10—C5	117.5 (3)
C3—C4—H4A	120.0	C9—C10—C5	125.0 (3)
			
C5—N1—C1—C2	−0.2 (5)	C6—C7—C8—C9	−0.7 (5)
N1—C1—C2—C3	−2.9 (6)	C7—C8—C9—C10	1.6 (5)
C1—C2—C3—C4	3.3 (6)	C6—N2—C10—C9	−0.1 (5)
C2—C3—C4—C5	−0.5 (5)	C6—N2—C10—C5	−178.9 (3)
C1—N1—C5—C4	3.0 (5)	C8—C9—C10—N2	−1.2 (5)
C1—N1—C5—C10	−175.6 (3)	C8—C9—C10—C5	177.6 (3)
C3—C4—C5—N1	−2.6 (5)	N1—C5—C10—N2	158.5 (3)
C3—C4—C5—C10	175.9 (3)	C4—C5—C10—N2	−20.1 (5)
C10—N2—C6—C7	1.0 (5)	N1—C5—C10—C9	−20.3 (5)
N2—C6—C7—C8	−0.6 (5)	C4—C5—C10—C9	161.1 (4)

### Hydrogen-bond geometry (Å, º)

**Table:**

*D*—H···*A*	*D*—H	H···*A*	*D*···*A*	*D*—H···*A*
N1—H1*A*···Br2	0.86	2.37	3.178 (3)	156
N2—H2*A*···Br1^i^	0.86	2.35	3.145 (3)	154
C1—H1*B*···Br1	0.93	2.83	3.611 (4)	143
C9—H9*A*···Br2	0.93	2.81	3.675 (4)	155
C6—H6*A*···Br2^i^	0.93	2.84	3.619 (4)	142
C4—H4*A*···Br1^i^	0.93	2.86	3.697 (4)	150
C3—H3*A*···Br1^ii^	0.93	2.85	3.677 (4)	149

Symmetry codes: (i) *x*, *y*+1, *z*; (ii) −*x*, *y*+1/2, −*z*+3/2.
